# Supplementary material for: Health problems and violence experiences of nurses working in acute care hospitals, long-term care facilities, and home-based long-term care in Germany: A systematic review
Source: PLoS One. 2021 Nov 18;16(11):e0260050. doi: 10.1371/journal.pone.0260050 (PMC8601565; doi:10.1371/journal.pone.0260050)
Supplement: S1 File — (DOCX) [file pone.0260050.s002.docx]

| **Author (year)** | **1. Was the sample frame appropriate to address the target**  **population?** | **2. Were study participants sampled in an appropriate way?** | **3. Was the sample size adequate?** | **4****. Were the study subjects and the setting described in detail?** | **5. Was the data analysis conducted with sufficient coverage**  **of the identified sample?** | **6. Were valid methods used for the identification of the condition?** | **7. Was the condition measured in a standard, reliable way for all participants?** | **8. Was there appropriate statistical analysis?** | **9. Was the response rate adequate, and if not, was the low response rate managed appropriately?** | **% (Yes)** |
| --- | --- | --- | --- | --- | --- | --- | --- | --- | --- | --- |
| 1. Aiken et al. (2012) | Y | Y | Y | N | Y | Y | Y | Y | N | 77.8% |
| 1. Diehl et al. (2020) | Y | Y | Y | Y | Y | Y | Y | Y | U | 88.9% |
| 1. Drupp & Meyer (2019) | Y | Y | Y | Y | Y | Y | Y | Y | Y | 100% |
| 1. Ehegartner et al. (2020) | Y | Y | Y | Y | Y | Y | Y | Y | Y | 100% |
| 1. Fischer et al. (2020) | Y | Y | Y | Y | Y | Y | Y | Y | U | 88.9% |
| 1. Frey et al. (2018) | Y | U | N | Y | Y | Y | Y | Y | U | 66.7% |
| 1. Gencer et al. (2019) | Y | Y | N | Y | Y | Y | Y | Y | U | 77.8% |
| 1. Grobe & Steinmann (2019) | Y | Y | Y | Y | Y | Y | Y | N | Y | 88.9% |
| 1. Kowalski et al. (2010b) | Y | Y | Y | Y | Y | Y | Y | Y | Y | 100% |
| 1. Lehmann-Willenbrock et al. (2012) | Y | Y | N | Y | Y | Y | Y | Y | Y | 88.9% |
| 1. Lindner et al. (2015) | N | Y | N | N | Na | U | Y | Y | U | 33.3% |
| 1. Lohmann-Haislah et al. (2019) | Y | Y | Y | Y | Y | U | Y | Y | Na | 77.8% |
| 1. Otto et al. (2019) | Y | U | N | N | Y | Y | Y | Y | U | 55.6% |
| 1. Paffenholz et al. (2020) | Y | N | Y | N | Y | N | Y | Y | U | 55.6% |
| 1. Raspe et al. (2020) | N | Y | N | Y | Y | Y | Y | Y | U | 66.7% |
| 1. Rothgang et al. (2020) | Y | Y | Y | N | Y | Y | Y | Y | Y | 88.9% |
| 1. Schablon et al. (2012) | Y | U | Y | N | Y | U | Y | Y | Y | 66.7% |
| 1. Schablon et al. (2018) | Y | Y | Y | N | Y | Y | Y | Y | Y | 88.9% |
| 1. Schmidt (2010) | Y | N | N | Y | Y | Y | Y | Y | Y | 77.8% |
| 1. Schmidt & Diestel (2011) | Y | U | N | N | Y | Y | Y | Y | U | 55.6% |
| 1. Schmidt & Diestel (2014) | Y | U | N | Y | Y | Y | Y | Y | U | 66.7% |
| 1. Skoda et al. (2020) | Y | N | Y | Y | Y | Y | Y | N | Na | 66.7% |
| 1. Vaupel et al. (2020) | Y | Y | N | Y | Y | U | Y | Y | N | 66.7% |
| 1. Vaupel et al. (2021) | Y | Y | Y | Y | Y | U | Y | Y | N | 77.8% |
| 1. Wagner et al. (2019) | Y | N | Y | N | Y | Y | Y | Y | U | 66.7% |
| 1. Weidner et al. (2017) | Y | Y | Y | Y | Y | U | Y | Y | U | 77.8% |
| 1. Weigl & Schneider (2017) | N | N | N | N | Y | Y | Y | Y | U | 44.5% |
| 1. Wirth et al. (2017) | Y | N | N | Y | Y | Y | Y | Y | Y | 77.8% |
| 1. Wollesen et al. (2019) | Y | Y | N | Y | Y | Y | Y | Y | U | 77.8% |

*Y=Yes, N=No, U=Unclear, Na=Not applicable*
