# Supplementary material for: Health problems and violence experiences of nurses working in acute care hospitals, long-term care facilities, and home-based long-term care in Germany: A systematic review
Source: PLoS One. 2021 Nov 18;16(11):e0260050. doi: 10.1371/journal.pone.0260050 (PMC8601565; doi:10.1371/journal.pone.0260050)
Supplement: S2 File — (DOCX) [file pone.0260050.s003.docx]

**PubMed search strategy**

Search terms used

1. Nurs* [All Fields]
2. „professional care“ [All Fields]
3. „professional caregiver“ [All Fields]
4. Health [All Fields]
5. Violence [All Fields]
6. „cross-sectional“ [All Fields]
7. Survey [All Fields]
8. German* [All Fields]
9. 2010/01/01:2021/01/11 [Date - Publication]
10. German [Language]
11. English [Language]

Search strategy (Boolean operators in cursive)

(1 *OR* 2 *OR* 3) *AND* (4 *OR* 5) *AND* (6 *OR* 7) *AND* 8 *AND* 9 *AND* (10 *OR* 11)
