# Supplementary material for: Health problems and violence experiences of nurses working in acute care hospitals, long-term care facilities, and home-based long-term care in Germany: A systematic review
Source: PLoS One. 2021 Nov 18;16(11):e0260050. doi: 10.1371/journal.pone.0260050 (PMC8601565; doi:10.1371/journal.pone.0260050)
Supplement: Attachment — Submitted filename: PLOS ONE paper review 5.docx [file pone.0260050.s004.docx]

**PONE-D-21-22976**

**Re: Health problems and violence experiences of nurses working in acute care hospitals, long-term care facilities or home-based long-term care: a systematic review.**

I almost felt that these was two systematic reviews in one. It could be “Health problems of nurses” and “Violence Experiences among nurses” I am not sure what is the relation between both? Why to include both under one systematic review? Furthermore, I have the following **Comments:**

**Title:** the word “or” should be “and” as both settings were reviewed by the authors. Also add “in Germany” under the title as the authors excluded studies outside Germany.

**Introduction:** should be rewritten and it will benefit from details explanation on the impact of workplace violence and its risk factors and why to include this topic under systematic review? What is the gap in the current literature in this regard? What is the aim of this systematic review?

Under introduction Line 60: the statement “In 2018, 19.4 million patients were treated in hospitals” Which hospitals?

Under introduction Line 77: “Theycan” should read “They can”

**Methods:**

Require major revisions as explained below:

**Search strategy:** Why only used PubMed and PubPsych? How about other database such as Medline, CINAHL, Web of sciences database? Explain in details Keywords and MESH. What are the Boolean operators used in this study? What are the participants used in the search? Did they include nurse practitioners, supervisors, and managers of nursing, ….etc? Authors should not used health insurance reports as these unreliable from scientific point of view. Only published studies can be used in Systematic Reviews.

**Inclusion and Exclusion criteria:** should be explained in detail. Why to include only cross-sectional studies? These subjected to bias. What are skilled nurses in Germany? Why they excluded longitudinal studies and studies outside of Germany?

**Quality assessment**: JBI checklist of prevalence studies is not appropriate to use in this review. The two independent reviewers should assess the included studies using the Critical Appraisal Skills Program (CASP) checklist (Critical Appraisal Skills Program (CASP) Checklist, 2016) in two phases. During the first phase, each reviewer read the title and abstract of all the citations retrieved and entered this information into a custom-designed database. In the second phase, full-text articles that met the inclusion criteria should be retrieved and reviewed. Pertinent information related to health problems and violence experiences among nurses should be collected and stored in the database.

The CASP used to evaluate the methodological rigor of each of the studies. Also use the Cochrane Collaboration ‘Risk of bias’ assessment tool across six domains of bias including selection, performance, detection, attrition, presorting and other (Higgins, Altman, & Sterne, 2011).

Furthermore, the inter-rater reliability should be assessed by Kappa statistical test. Then authors should report the Kappa level?

**Study selection:** What is the definition of violence included in this Systematic Review (SR)? Type of violence? Did the authors explored whether the reporting of 29 studies associated with being Cochrane review?

**Data extraction**

Authors should perform data extraction on a random sample of the included systematic Review (SR) which were selected using the random number generator. Sampling should be stratified so that the proportion of Cochrane reviews in the selected sample equaled that in the total sample

To minimize errors in the remaining sample of SR, one author verifies the data for these items in all SR. Also, one author reviewed the free text responses of all items with an “Other (please specify)” option. Responses should be modified if it was judged that one of the forced-choice options was a more appropriate selection.

**Data synthesis**

Data synthesis should involve a mixture of descriptive summaries of the included methodological research papers. Data extracted from research articles that described a summary of measures (odds ratios, the difference in means, incident rate ratios) should be grouped and analyzed by study design. From this analysis, you should prepare a descriptive analysis of the included studies. Authors did not explain their data synthesis.

**Results:**

**Figure 1 PRISMA Flow chart has many errors, and it is very confusing. For example, f**ull text article in figure 1 were excluded with reasons 20….etc., these did not add up into n=37. Also 8 studies are missing to come down from 37 to 29. The total studies included under manual search were 22 and if these included in the final 29 we left with only 7 and how many of these either from Pubmed or PubPsych? Please clarify.

Why to include studies with setting unspecific as reported under Table 1. These should be excluded from this SR as it contradicts with the aim of the study and the title of this SR.

Tables should be revised as per the above comments

**Discussion:** the statement “this is the first review” is very strong and authors unaware of unpublished work. I suggest deleting it

**Limitations of the study:** should explain in details and logical ways. Personal, misinterpretation, publication biases are possibilities in this SR.
